# Low-Dimensional Structures for Smart Materials and Composites: Preparation, Properties and Applications

**DOI:** 10.3390/ma16175743

**Published:** 2023-08-22

**Authors:** Federico Cesano

**Affiliations:** Department of Chemistry, Turin University & INSTM-UdR Torino, 10125 Torino, Italy; federico.cesano@unito.it; Tel.: +39-011-670-7548

**Keywords:** 0D, 1D, 2D, low-dimensional structures

## Abstract

The Special Issue covers low-dimensional structures or systems with reduced spatial dimensions, resulting in unique properties. The classification of these materials according to their dimensionality (0D, 1D, 2D, etc.) emerged from nanoscience and nanotechnology. One review and eighteen research articles highlight recent developments and perspectives in the field of low-dimensional structures and demonstrate the potential of low-dimensional systems in various fields, from nanomaterials for energy applications to biomedical sensors and biotechnology sector.

## 1. Introduction

Low-dimensional structures refer to materials or systems that have unique characteristics due to their reduced spatial dimensions in physical space. The reduced dimensionality can result in quantum confinement effects and increased surface-to-volume ratios, leading to new properties, as compared to their bulk counterparts [1]. The classification of materials according to their dimensionality (0D, 1D, 2D, etc.) resulted from the development of nanoscience in the late of the 20th century.

**Figure 1:**
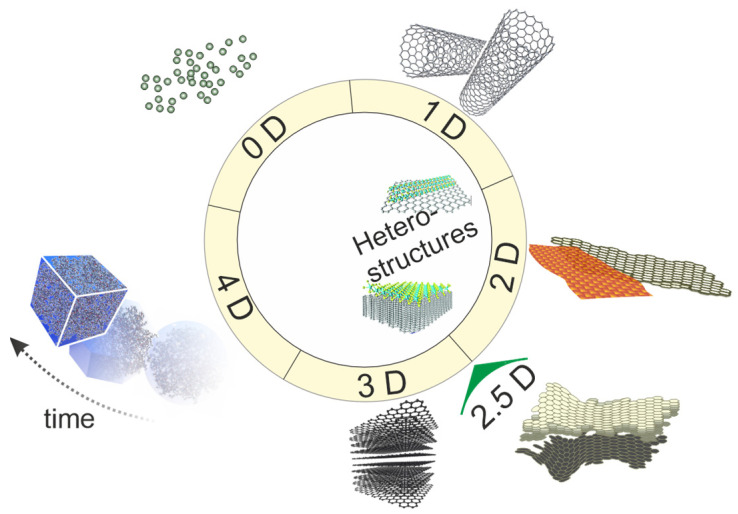
Classification of structures, based on their dimensionalities. The representation includes heterostructures that often involve 0D/1D, 1D/2D hybrid structures, etc.

Materials can be classified according to their dimensionality (Figure 1), as follows:

- Zero-dimensional (0D)-materials: all 3 dimensions are confined at the nanoscale (1–100 nm). For example: quantum-dots, nanoparticles;
- One-dimensional (1D)-materials: dimensions are elongated along **a** single axis. For examples nanotubes/nanowires/nanorods;
- Two-dimensional (2D)-materials: thicknesses are at the atomic scale, but lateral sizes can extend over. For example: graphene and transition-metal-dichalcogenides;
- Three-dimensional (3D)-materials: sizes are well-defined in all dimensions, and they are not restricted to specific dimensionality.

Some layered structure materials can exhibit stronger interactions between layers, leading to the entire system a 2.5D characteristic [2]. Such systems display features that are not purely confined into a planar, but they do not have a 3D domain. 4D-structures usually refer to systems exhibiting changes in their physical properties (i.e., optical or any other wave and field phenomena) or configurations over time [3]. These structures are often designed or engineered to respond to external stimuli in a predictable and controlled manner. The term “4D structures” can be more commonly used in the field of metamaterials, energy materials, etc. [4].

## 2. The Special Issue

This Special Issue comprises one review and eighteen research articles, highlighting some recent improvements and perspectives in the field of low-dimensional structures.

One of the most productive research areas is certainly the electrical properties of composites containing conductive fillers (i.e., carbon black, CNTs, graphene). This extensive research comes from the relatively cheap/easy processability of polymers when added with nanofillers, and the possibility to impart to the material electrical/sensing properties [5]. Beltran *et al.* [6] focused on polylactic acid (PLA) polymer reinforced with CNTs at different weight percentages (CNTs 0.25–5 wt.%). The authors investigated the direct current (DC) and radio frequency (RF) microwave electrical conductivities of composites and calculated percolation thresholds using classical models. The theoretical determination of the electrical percolation threshold revealed that it occurred at ~1 wt.% and ~0.25 wt.% for DC and for the microwave regime, respectively. Among the Mamunya, McLachlan, and generalized effective medium (GEM) models, the latter one showed a good fit with both the experimental DC and RF regimes. Specifically, the Mamunya model exhibited similarities with the experimental DC/RF conductivity at higher CNT concentrations, as expected from the percolation law. Moreover, the McCullough model had limitations, including chain-breakage statement, leading to reduced RF-conductivity at high CNT loading. Conversely, through-plane electrical conductivities of composites were successfully predicted by the GEM model, demonstrating a close agreement with experimental results. In conclusion, the paper holds significant implications for the development of conductive polymer composites with improved electrical properties for various applications. Maharjan *et al.* [7] developed a flexible piezoresistive sensor utilizing a graphene/PVDF nanocomposite. Interestingly, this sensor was designed to be flexible and to work as an accelerometer for detecting low-frequency low-amplitude vibrations. The sensor featured a cantilever beam with a proof mass positioned at its free end. When exposed to vibrations, the proof mass responded dynamically, oscillating up/down and generating a distinct electrical signal, which was measured as a ΔR, expressed as a response percentage relative to the detected acceleration. The authors demonstrated capabilities to detect acceleration values up to 8 g within 20–80 Hz. Remarkably, the authors noticed that this study showed for the first time piezoresistive effects of graphene-based nanocomposites in developing a low-amplitude accelerometer. To summarize, this flexible piezoresistive accelerometer presents a highly adaptable and innovative solutions for effectively detecting vibrations in industrial processes. Sepehri *et al.* [8] adopted the eddy-current and impedance spectroscopy to detect changes in rubber properties after thermal aging. The authors investigated ethylene-propylene-diene-monomer (EPDM) rubbers containing carbon black and graphene, using both eddy-current sensing and electrical impedance measurements for electromagnetic analysis. Measurements were taken before and after the aging, by measuring the electrical properties in rubber samples by varying the CB/graphene contents. Replacing graphene with CB in the EPDM rubber formulation resulted in an enhanced eddy-current and electrical impedance (Z)-responses. For Z-measurements, a significant difference between non-aged and aged samples was observed, with graphene concentration causing variations in both permittivity and conductivity. Interestingly, while the eddy-current response increased with thermal aging, Z-measurements showed reductions in both conductivity and dielectric response. The authors attributed this mismatch to the probing electric field direction differences with respect to the mechanical load during thermal aging. This fact suggested that thermal-aging combined with applied mechanical loading can create an anisotropy in conductivity/dielectric response. The results obtained from the eddy-current method make it feasible to assess a non-destructive method to evaluate properties in CB/EPDM rubber materials.

The interest towards low-cost/mass-production of low-dimensional-carbons and applications is still extremely active. Ostermann *et al.* [9] developed a sustainable process to produce graphene oxide (GO) using a method consisting in three-stage electrochemical exfoliation. XRD analysis, XPS and Raman spectroscopies confirmed the fast exfoliation rates, resulting in significantly higher conductivities compared to GO produced via the commonly used modified Hummers’ method. The proposed GO powder protocol indicated a linear relation between the amount of educt surface and the volume of electrolyte, supporting inexpensive large-scale production up to the Kg amount. The authors tested the method for three different volume-to-area ratios for electrolyte/graphite precursors and observed near-linear scaling. The obtained conductivity was found to be 4-fould after mild thermal reduction, offering energy savings compared to traditional production processes. In summary, the proposed strategy opens opportunities for industrial applications, where cost and environmental impact are critical considerations. Obayomi *et al.* [10] reviewed recent methods utilizing graphene-based nanomaterials for removing phenol from palm-oil-mill-effluent (POME). Phenol compounds are a major concern, due to their presence and harmful effects on ecosystems and human health. The authors investigated various aspects, such as material adsorptive properties, experimental parameters, isotherms/kinetic models, formation mechanisms, and the efficacy of graphene-based materials as adsorbents. In conclusion, the use of these nanocomposites takes advantages from a high-surface-area, mechanical strength, and chemical/physical properties, resulting good phenol adsorption capabilities. Kinetic studies indicate that GO-based nanocomposites typically demonstrate a pseudo-second-order reaction for phenol adsorption, highlighting their rapid and efficient removal of phenol from wastewater. Flores-López *et al.* [11] explored the use of reduced-graphene oxide (rGO) aerogels as effective extractants for the separation of UV-benzotriazoles, which are known to be water micropollutants causing serious threats. Graphene-based materials have been identified as potential candidates to assist in solid phase extraction (SPE) processes. In this context, the authors focused on rGO aerogels as candidate materials that could overcome the limitations of conventional graphene-based materials. The authors synthesized rGO aerogels with varying content and structural order of graphene domains. The authors illustrated some advantages of aerogels over traditional graphene-based materials, including the composition and tunable porous properties as solid extractants for UV-benzotriazoles. Parmar *et al.* [12] investigated the CNT impact in the intercalation capacity of binder-free manganese vanadium oxide (MVO)-CNT composite electrodes for sodium-ion-batteries (SIBs). The authors analyzed the cathode-electrolyte-interphase (CEI) layer formed during cycling and observed variations in CEI distribution based on the CNT wt%. The fading capacity of MVO-CNTs observed by the authors was found to be associated with the dissolution of the Mn_2_O_3_ phase, which was observed in electrodes with low CNTs content, causing disruptions in their tubular topology. The study highlighted the crucial role played by CNTs in CEI formation and phase stability, as they provided a conductive network that facilitated the deposition of MVO. The optimization of CNTs content resulted in an improved electronic structure of the electrode, leading to a stable and uniform phase during charge-discharge cycles. In summary, the research conducted by Parmar sheds light on the role of carbon nanotubes in improving the performance and stability of MVO-based electrodes for sodium ion batteries. The findings contribute to the growing understanding of the intercalation mechanisms in these battery systems and pave the way for advancements in energy storage technologies.

Polymers are very versatile systems, whose linear/branched chains can be adapted to create porosity or accommodate metal nanoparticles to be used as SERS supports. Kovylin *et al.* [13] fabricated 2-mm thick porous polymer monoliths using visible light-induced radical polymerization of oligocarbonate dimethacrylate (OCM-2) with 1-butanol as a porogenic additive. The authors found that monolithic polymers with open and closed pores (up to 100 nm) were formed when 1-butanol content was as low as 20 wt.% and that the porous texture was found to consist of a system of holes, referred to as hole-type pores. When 1-butanol content exceeded 30 wt.%, interconnected pores (up to 10 microns) were formed, due to covalently bonded polymer globules, known as interparticle-type pores. The transition range of 1-butanol concentrations between 20 and 30 wt.%, exhibited both hole-type and interparticle-type structures, along with honeycomb structures of interconnected polymer globules. The significance of the paper lies in developing biocompatible, non-cytotoxic porous polymers for osteoplasty materials, offering tailored interactions with different cell populations. Rodrigues *et al.* [14] discussed the development of thin-films composed of nanohybrid-conjugated polymers (CPs) and metallic nanoparticles for potential applications as SERS sensors of pesticides. The authors developed thin solid films using aqueous dispersions containing nanoparticles made of small-conjugated polymer (NCP) and triangular and spherical silver nanoparticles, which were blended with the NCP-based films to enhance their SERS properties. Interestingly, the authors showed that the shape of the Ag NPs influenced the adsorption between the NCP and the metal surface, with a perpendicular adsorption between the NCP chains and the surface of triangular Ag NP and that the combination of CPs and different-shaped Ag NPs altered the morphological and electronic properties of both materials. The paper contributes to the understanding of how copolymers of polyfluorene and thiophene, along with metallic nanoparticles of varying dimensions and geometries, behave.

Inorganic low-dimensional-structure is an extremely active and wide research field, as testified by the following case studies. These low-dimensional-structures can be tied into pillars and other geometrically organized macrostructures [15]. Manseki *et al.* [16] presented an example of the structure recognition and narrow-bandgap properties of assembled SnO/SnO_2_ nanoparticles, as derived from the thermal treatment in air of Tin(II) oxalate. The authors observed the simultaneous formation of SnO/SnO_2_ nanoparticles with sizes of 20–30 nm arranged into nanostructured rods, as obtained during the heating step (350–600 °C range). In the paper, it is shown that the introduction of Sn (II) was achieved by adjusting the concentration of Sn (II) salt during precursor synthesis at the maximum calcination temperature (600 °C). The resulting SnO_x_ nanorods, exhibited photoabsorption properties with a lower bandgap of 2.9~3.0 eV (associated with SnO) and a higher bandgap of around 3.5~3.7 eV (typical of SnO_2_). These 1D SnO_x_/SnO_2_ hybrid structures provide insights into the development of photo-functional SnO nanomaterials for new material designs in photoenergy conversion systems. Grabarczyk *et al.* [17] discussed the practical application of an eco-friendly **electrochemical** sensor for the Cd (II) detection using low-dimensional-structures, spherical glassy carbon microparticles, and multiwall carbon nanotubes. The sensor was modified with a bismuth film and employed anodic stripping voltammetry for Cd(II) determination. The authors investigated factors affecting sensitivity, and obtained optimal values. Importantly, the sensor demonstrated minimal interference from other ions. To validate its applicability, the sensor was tested with certified reference materials and real river water samples, yielding accurate and reliable results. Overall, this eco-friendly electrochemical sensor holds interest for efficient and precise detection of Cd(II) in the environment. Buryi *et al.* [18] studied the influence of Mo on various properties of ZnO rods grown as free-standing nanoparticles. Several experimental techniques were adopted to investigate the impact of Mo on electronic states, crystalline structure, morphology, phase composition, luminescence, and defects in the ZnO rods. The authors found that exciton and defect-related bands in ZnO rods experienced a remarkable drop upon annealing in air with Mo doping. In addition, it was observed that increasing the Mo doping level from 20% to 30% led to the formation of dominating molybdate phases with a consequent decrease in the number of ZnO nanorods. In summary, the paper underscores a research about the significance of Mo in shaping the properties of ZnO rods and provides valuable insights into the potential applications of Mo-doped ZnO materials. Lupi *et al.* [19] introduced an innovative electrodeposition process aimed at depositing fiber-optic-Bragg-gratings (FGBs) with a Ni coating The authors investigated the evolution of the coating properties (i.e., grain-growth and oxidation). This aspect was of utmost importance as it provided insights into the behavior of the sensors under extreme temperature conditions. The authors investigated variations in the reflected spectrum for the understanding of the overall performance and structural integrity of the FGBs. The results presented a prospect for employing these sensors in industrial applications, where extreme temperatures are adopted. Abdullah *et al.* [20] investigated the influence of the calcination temperatures and durations in the synthesis of iron-oxide nanoparticles. Two methods were adopted: *Phoenix dactylifera* L. (PDL) extract as the reducing agent (green method), and NaOH (chemical method). The authors showed that the calcination temperature and time significantly affected the degradation of the active substance (polyphenols) and the final structure of iron-oxide nanoparticles. Lower calcination temperatures and shorter durations yielded NPs with smaller sizes, and enhanced antioxidant activities. Notably, the study highlighted the importance of green synthesis of iron oxide nanoparticles using PDL extracts due to their antioxidant and antimicrobial properties. Iron-oxide nanoparticles, synthesized via green synthesis demonstrated remarkable antioxidant and antibacterial properties. As a result, they hold great promise as a potential alternative to conventional antibiotics for antibacterial drug applications. Molecular beam epitaxy (MBE) is a widely used method for engineering low-dimensional-materials. Dziwoki *et al.* [21] introduced a novel extension to MBE by incorporating an external magnetic field (MF) during epitaxial growth. The MF-assisted epitaxial growth was conducted under ultra-high vacuum conditions using sample holders capable of generating in-plane/out-of-plane MF. The authors focused on ultra-thin epitaxial magnetite films grown on MgO (111) substrates and observed that the external MF induced changes in the growth morphology. Simulations based on a 1D model provided quantitative determinations of the anisotropies involved, showing that in-field deposition contributed to an additional perpendicular uniaxial anisotropy, which strongly outweighed the magneto-crystalline anisotropy. In conclusion, a moderate magnetic field of 0.1 T induced significant effects on the magnetic anisotropy, suggesting a magneto-strictive influence rather than direct effects on growth kinetics, which can be adopted for tailoring magnetic of more complex epitaxial heterostructures. Kaydashev *et al.* [22] presented a method involving the exploitation of surface acoustic waves (SAWs) to simultaneously probe the altered temperature and electrical conductivity of a hybrid Au nanoparticles-VO_2_ film composite heated and exposed by laser light. The authors reported a resolution of ±0.1 °C, which is comparable with the best existing techniques to study small objects and surfaces. In conclusion, the method offers a way to characterize photothermal effects in nanosystems/metasurfaces/optoelectronics. Tumurbaatar *et al.* [23] adopted adsorption methods for CO_2_ capture and the modification of mesoporous silica materials to improve their CO_2_ adsorptions. A new derivative containing an electron-rich condensed aromatic structure, was applied to modify SBA-15/SBA-16/KIT-6 silicates. The physical-chemistry properties of the initial/modified materials were studied by the authors, and the CO_2_-uptake was measured under dynamic conditions. Interestingly, the polar-groups presence and electron-rich fragments in the grafted agent’s structure favored CO_2_ capture, even in the presence of water vapor. The results suggested that the modified silicas exhibit different pore structures and surface properties for efficient CO_2_ capture.

Biosensing and biotechnology become target sectors in specific applications using low-dimensional-systems. For example, Jang *et al.* [24] developed an electrical biosensor designed for Zika-virus (ZV) infection detection. The biosensor was based on a truncated DNA aptamer (i.e., short nucleotide sequences that maintain a tertiary structure), immobilized on an interdigitated Au micro-gap electrode. Generally, DNA aptamers are considered to have relatively low-dimensional structural characteristics when compared to more complex biomolecules like proteins or large nucleic acids, despite their ability to adopt specific three-dimensional conformations for target binding. The authors used alternating current electrothermal flow (ACEF) technique. The quick detection in diluted serum samples was achieved by the authors, who attributed the efficiency of the biosensor to the ACEF technique that induced an electrothermal effect on the electrode, resulting in a local temperature gradient in the microfluid that reacts with the electrode. Overall, the biosensor could help in the development of a fast and reliable virus detection platform.

Microencapsulated extracts can be considered a low-dimensional system in the biomedical science because the encapsulation process reduces the dimensionality, being the extract confined within the polymer shells. In this context, Navolokin *et al.* [25] developed microencapsulated and unencapsulated Gratiola officinalis extract on breast cancer SKBR-3 and cervical cancer HeLa cells. The authors reported that the microencapsulated extract exhibited significantly higher effectiveness, causing 100% death of breast cancer cells and 34% death of cervical cancer cells, while also preventing autophagosome formation in both cultures. These findings demonstrate that microencapsulation is an effective strategy for delivering extracts to tumor cells.

I sincerely hope that the published contributions can help readers’ knowledge in the field of low-dimensional systems, offering inspiration for new research studies. I would extend my sincere gratitude to the authors for their precious contributions, the referees for their shrewd and essential remarks, and the Editorial staff for the unparalleled support.
